# Experimental investigation of the mechanical properties of mud shale under water-bearing conditions and its applications

**DOI:** 10.1038/s41598-022-13476-8

**Published:** 2022-06-08

**Authors:** Xinxin Fang, Hong Feng, Fengling Li, Hao Wang

**Affiliations:** 1 China Coal Research Institute, Beijing, 100013 China; 2 China Coal Technology & Engineering Group Xi’an Institute, Xi’an, 710077 Shanxi China; 3 Oilfield Technology Service Company of Xinjiang Oilfield Company, PetroChina, Karamay, 834000 China; 4 Research Institute of Petroleum Exploration and Development, Beijing, 100083 China

**Keywords:** Natural hazards, Solid Earth sciences, Energy science and technology, Engineering

## Abstract

Mud shale, used in drilling engineering, is prone to hydration and expansion, resulting in creep deformation that leads to wellbore shrinkage and pipe sticking incidents. Studying the creep characteristics of mud shale is significant for designing a reasonable well structure and determining the lower limit of drilling fluid density. The influence of moisture content on rock strength and creep mechanical properties were studied using water absorption, uniaxial compression, and creep tests. Test results show that with an increase in the moisture content, the mud shale was damaged and softened; moreover, the elastic modulus decreased with increase in moisture content. Under the same load level, the instantaneous strain increased with increasing moisture content. Under different loading stresses, the creep of the rock had nonlinear characteristics, which could be divided into three different creep stages: attenuation, second, and accelerated creep. A new improved creep model based on the Nishihara model was established to describe the accelerated creep characteristics of mud shale under different moisture contents. The ageing degradation and water-bearing weakening effects were introduced. The Levenberg–Marquardt nonlinear least-squares method was applied to invert the creep parameters. The results show that the simulated creep curves, generated using the new creep model, conform to the experimental ones. The relationship between the drilling fluid density and wellbore shrinkage ratio can be defined using this model; it provides a reference for reasonably determining the drilling fluid density.

## Introduction

Mud shale formations are often encountered during drilling. After the formation is drilled, the drilling fluid may come into contact with the mud shale; this causes a hydration reaction, which reduces the strength of the rock and causes the wellbore to collapse or shrink in diameter^1–3^. Borehole wall instability can be classified into wellbore section shrinkage, tensile breaking damage, and wellbore collapse, which are common in mud shale formations^4–6^. Thus, wellbore stability is almost synonymous with mud shale stability. Creep deformation is a typical characteristic of mud shales. When mud shale hydration expands to produce creep deformation, the open hole wall will cause shrinkage damage and a series of incidents, such as borehole instability, sticking, squeezing, and destruction of the casing after cementing, which results in significant economic losses to the drilling process^7–9^. A suitable evaluation method for borehole wall instability caused by illite and montmorillonite mixed layers has not yet been found. The study of creep characteristics of water-rich mud shale with time can help determine the rheological law of hard brittle shale under water-bearing conditions, and provide a reference for determining the drilling mud density, thereby ensuring the stability of mud shale walls.

Experimental studies of rock creep have been conducted since the 1930s. Griggs conducted a series of creep experiments on sandstone and mud shale and found that creep will occur when a load on such rocks reaches 12.5–80% of the failure load^10^. Huang performed uniaxial creep tests on argillaceous siltstone under different water content states and established mathematical relationships between creep modulus and water content^11^. Hawkins and McConnell conducted tests on 35 groups of sandstones and found a negative exponential relationship between moisture content and strength; that is, strength and deformation characteristics gradually decreased with an increase in moisture content^12^. Rock creep constitutive models were studied using various theories including empirical, element model, viscoplastic model, and damage model theories. The component theoretical model describes creep behaviour by combining basic components (including the Hooker, Newton, and Saint Venant bodies) in series or parallel. Currently, Kelvin, Burgers, and Nishihara models are widely used. The Nishihara model comprises a three-parameter H–K model with viscoplastic components in series, which has the advantage of a more comprehensive expression of creep deformation, compared with the Kelvin and Burgers models and is most often used in creep experiments and numerical simulations^13–15^. Singh and Mitchell used the Nishihara model to conduct a viscoelastoplastic rheological analysis of deep plagioclase amphiboles. The fitted curves were in good agreement with the creep and relaxation curves obtained from the test^16^. Grgic considered creep in coal to behave as a viscoelastoplastic body that satisfies the Nishihara model and obtained a model that can predict the deformation of a coal pillar^17^. In terms of water damage creep, Chen XY introduced transient elastic damage and long-term creep damage variables into the Burgers model and established a constitutive creep equation considering the water damage effect^18^.

Previous studies concerning the influence of water on the mechanical properties of rocks have mainly focused on the strength and creep tests under the two extreme water-bearing states: dry and saturated. The mechanical properties of creep of mud shale, mainly composed of illite and montmorillonite, under the combined action of load and water in a water environment, have been particularly neglected. Present research on the Nishihara model and moisture content creep mainly focuses on the steady-state creep stage. In contrast, research on the accelerated creep stage is insufficient, and the traditional Nishihara model cannot reflect the characteristics of nonlinear accelerated creep.

**Figure 1:**
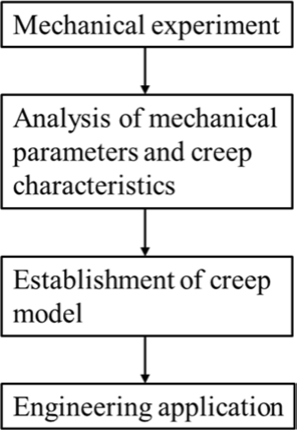
Flowchart of study.

Therefore, based on the aforementioned analysis, this study uses mud shale from the western China to conduct conventional uniaxial compression strength and creep tests in a continuous water environment for specimens with different moisture contents. Based on the creep mechanics test, the Nishihara model was further improved by introducing the ageing degradation and water reduction effect, starting from the perspective of a nonlinear viscous pot. A new component model that reflects the creep acceleration behaviour of mud shale was proposed and verified by test results. It can provide theoretical guidance for shaft wall stability and safe production under complex underground groundwater and in-situ stress conditions. The basic steps of this study are as shown in Fig. 1.

## Samples

**Figure 2:**
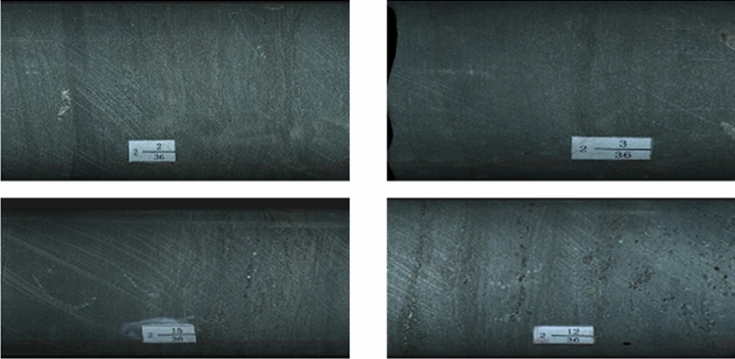
Mud shale samples.

The mud shale studied in this work comes from the western China, as shown in Fig. 2. When drilling cores using the hydraulic drilling method, the rock samples are easily disconnected along the bedding plane, making it difficult to reach the required length for the experiment; thus, it is difficult to prepare rock samples. The rock was therefore frozen and then drilled with kerosene. The drilling rate was controlled during the drilling process to obtain a standard rock sample that met the requirements of the experiment. According to the standards recommended by the International Society of Rock Mechanics^19^, the core was processed into a standard cylindrical sample with dimensions of φ25 mm × 50 mm.

**Figure 3:**
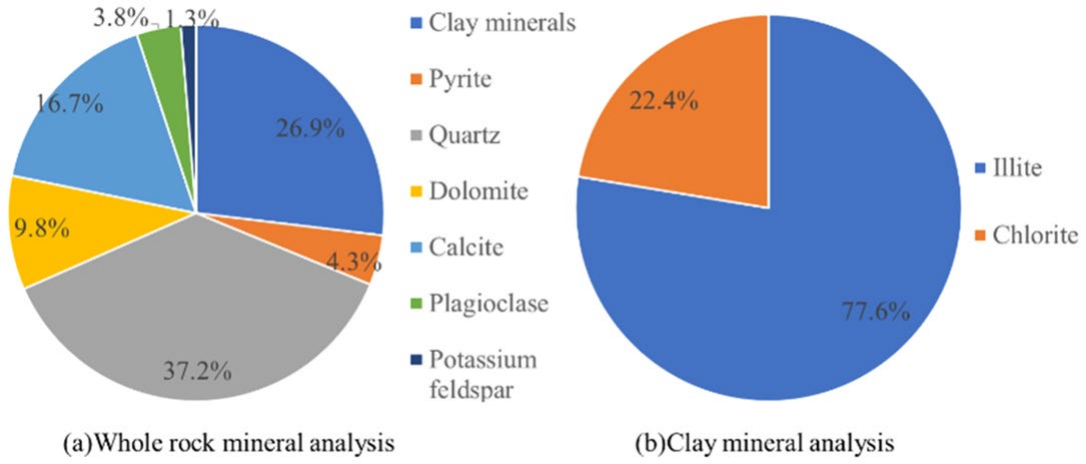
Mineral composition of shale.

As shown in Fig. 3, the main mineral components of the mud shale in the western Sichuan Basin were quartz, calcite, and clay minerals with a content of 26.9%, 16.7%, and 26.9%, respectively.

## Test methods

### Test instrument

**Figure 4:**
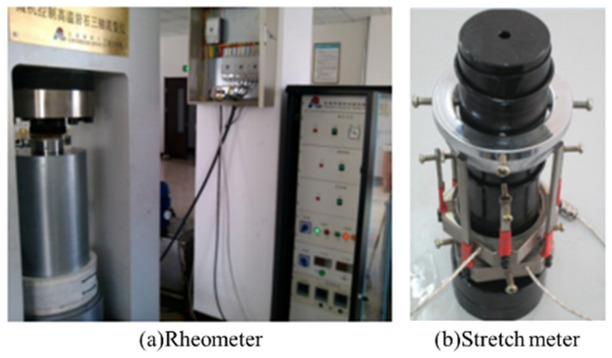
RLW-2000 rheometer and extensometer.

The RLW-2000 triaxial rheometer controlled by a microcomputer is a multifunctional rheometer developed by Changchun Chaoyang Testing Machine Factory, which can perform uniaxial and triaxial compression tests and creep seepage tests of rocks. The control system is an ideal controller with a German DOLI all-digital servo controller, which can realise closed-loop control of the test force, deformation, displacement, and smooth switching of the three control modes. The rheometer and extensometer after installation are shown in Fig. 4.

### Test process

There are two main creep loading methods: single-stage loading and step loading. In this experiment, the aforementioned electro-hydraulic servo test machine system was adopted, and a stepwise increasing load was applied to the same specimen by stepwise incremental loading. To study the creep response of rocks under the same stress state, the stress level was designed according to the uniaxial compressive strength obtained in the experiment. On this basis, the load was applied step-by-step on the same specimen in increasing order. Considering the low uniaxial compressive strength of the saturated sample, the initial creep stress was set as 16 MPa. An axial preload was first applied to the specimen; it was then slowly loaded to the rated load. Loading was stopped at this stage. The pressure was maintained constant, the instantaneous strain was recorded, and its displacement was continuously observed. After the entire creep process, the sample entered the next stage. The creep time of each stage was designed to be 24 h until creep failure of the sample occurred. In the initial stage of the experiment, the creep rate changed significantly, and the time interval of recording was small. After a certain period, when the creep strain rate tends to be steady, it was recorded every 1–2 h. In terms of experimental data processing, considering that rock creep is nonlinear and does not satisfy the principle of linear superposition, the data were processed using Chen’s loading method. To obtain the curve of the accelerated failure stage of creep and avoid brittle failure of the specimen in the process of applying excessive instantaneous load, the stress increment gradient was adjusted at the later stage of loading and reduced to half the initial design value until the accelerated creep sample was destroyed under the action of the last level of load.

### Water absorption test and determination of moisture content

Water absorption tests were performed on mud shale before the strength and creep tests to obtain the creep mechanics, deformation, and failure characteristics of mud shale under different water contents and stress states. During the experiment, the specimen was first baked at 105 °C for 24 h, after which the mud shale sample was considered to be in a dry state (moisture content of 0%). The dry specimen was then removed and cooled to room temperature; it was weighed and quickly immersed in a container filled with distilled water. The specimen was removed every 30 min within the first 10 h of immersion, and the surface moisture was wiped off with a wet cloth. The specimen was weighed on a high-precision balance, and weighed in the same way every 1 h after immersion. The moisture content $w$ of the rock after soaking for a certain time was calculated using the following formula:

1 $$ w = \frac{{\left( {M_{t} - M_{0} } \right)}}{{M_{0} }} \times 100\% $$

where M_t_ is the mass of the soaked rock specimen at time t and M_0_ is the mass of the dry rock specimen.

In this experiment, five different moisture contents were designed: 0% (dry state), 0.6%, 1.8%, 2.6%, and 3.6% (saturated state). The parameters of some experimental samples are listed in Table 1.

**Table 1:** Experimental sample parameters.

Sample Number	Moisture content/%	Mass before soaking/g	Mass after soaking/g	Length/mm	Diameter/mm	Density/g cm^−3^
X-1	0.0	318.46	–	50.31	25.12	2.58
X-2	0.6	320.52	321.92	50.28	25.09	2.56
X-3	1.8	343.21	349.39	50.37	25.16	2.61
X-4	2.6	349.28	358.36	50.26	25.15	2.59
X-5	3.6	338.55	350.74	50.27	25.05	2.60

## Experimental results and discussion

### Effect of moisture content on the mechanical properties of mud shale

**Figure 5:**
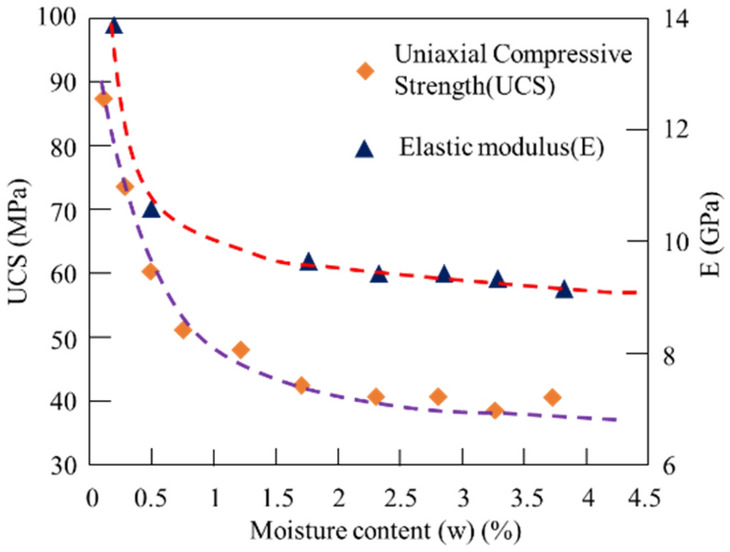
Relationship between rock strength parameters and moisture content of mud shale.

This study conducted multiple sets of uniaxial compressive strength experiments of mud shale under different moisture contents. Figure 5 shows the experimental results of the uniaxial compressive strength of mud shale under different moisture contents. The maximum uniaxial compressive strength of mud shale under dry condition is 89.510 MPa, whereas the compressive strength of ones under saturated condition is 39.67 MPa. This shows that the rock was damaged and softened with an increase in moisture content. Similarly, the elastic modulus decreased as the moisture content increased from 14.353 to 9.272 GPa. Equation (2) is obtained through nonlinear fitting, which is the softening equation of the uniaxial compressive strength (UCS) and elastic modulus (E) of mud shale with moisture content, where $w$ is the moisture content of the rock:

2 $$ \begin{aligned} & \sigma_{{\text{c}}} = 49.853*{\text{exp}}\left( { - 2.523*{\text{w}}} \right) + 39.657 \\ & {\text{E}} = 5.086*{\text{exp}}\left( { - 1.896*{\text{w}}} \right) + 9.267 \\ \end{aligned} $$

### Effect of step loading on creep characteristics of mud shale

**Figure 6:**
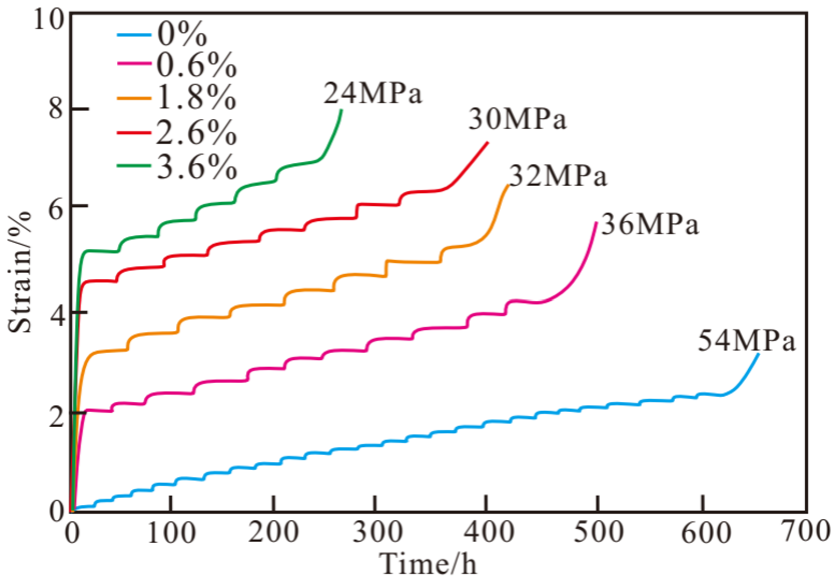
Creep curves of mud shale with different moisture content under step loading.

Based the experiment data, the whole process curve of mud shale with different moisture content under different load levels could be achieved (Fig. 6). Mud shale samples exhibit creep characteristics, which show that the deformation increases with time under a certain stress level. The instantaneous strain increased with increasing moisture content at the same load level. The higher the moisture content, the lower the load level reaching the acceleration stage. The sample with 3.6% moisture only needs 24 MPa to enter the accelerated stage. However, the sample with 0% moisture content needs 54 MPa to reach the accelerated stage. The creep deformation of mud shale increases with increasing moisture content. As can be seen from Fig. 6, under the same external loading conditions, the creep deformation of the dry rock sample was the smallest, that of the rock sample with 0.6% water content was slightly larger, and that of the samples with 3.6% water content was the largest.

## Establishment and application of improved creep constitutive model considering ageing and hydration damage

### Creep constitutive model considering the damage

According to the nonlinear creep characteristics of mud shale with different moisture contents, a constitutive creep model with ageing and hydration damage of rock in a water-bearing state can be established, which can reflect instantaneous elastic deformation, attenuation creep, steady creep, and accelerated creep. As can be seen from the test results, the creep property of water-bearing rock is related to the moisture content, stress state, and loading time. This study proposed a constitutive creep model that considers ageing and hydration damage based on the Nishihara model^20,21^.

#### Damage variables

The elastic modulus, strength and viscosity of rock usually decrease with time. Therefore, considering this degradation, the damage variables are defined as follows:

3 $$ D_{t} = 1 - \frac{{E_{t} }}{{E_{0} }} $$

where E_0_ is the initial elastic modulus; E_t_ is the elastic modulus at any time.

Based on the damage theory, damage is expressed as the ratio of the material defect area to the total effective bearing area of the material:

4 $$ D = \frac{{S_{0} - S_{w} }}{{S_{0} }} $$

where $S_{0}$ is the effective bearing area and $S_{w}$ is the effective bearing area in the damaged state. Therefore, the damage variable of the strength of the water-absorbing mud shale is

5 $$ D_{w} = 1 - \frac{{E_{w} }}{{E_{0} }} $$

Based on the elastic modulus varying with moisture content, the damage equation of the elastic modulus can be derived as follows:

6 $$ D_{w} = 0.413 - 0.413*{\text{exp}}\left( { - 1.586{\text{*w}}} \right) $$

The definition of long-term creep damage is based on the variation in the viscous modulus. The rock was considered to be in a dry state without damage, and its viscous modulus was ${\text{E}}_{{{\text{v}}0}}$. Thus, the creep damage variable can be defined as

7 $$ D_{c} = 1 - \frac{{{\text{E}}_{{\text{v}}} }}{{{\text{E}}_{{{\text{v}}0}} }} $$

Based on the creep modulus varying with moisture content, the damage equation of the creep modulus can be obtained as follows:

8 $$ D_{c} = 0.629 - 0.629*{\text{exp}}\left( { - 1.455{\text{*w}}} \right) $$

As could be seen in Table 2, the creep damage increased with increasing moisture content, the damage value was 0 in the dry state. However, when the mud shale sample reached the saturated water content, the damage approached the peak 0.628.

**Table 2:** Creep damage corresponding to different moisture content.

Moisture content (w/%)	Elastic damage (D_w_)	Creep damage (D_c_)
0.0	0.000	0.000
0.6	0.254	0.366
1.8	0.389	0.583
2.6	0.615	0.616
3.6	0.626	0.626

### Improved creep model

**Figure 7:**
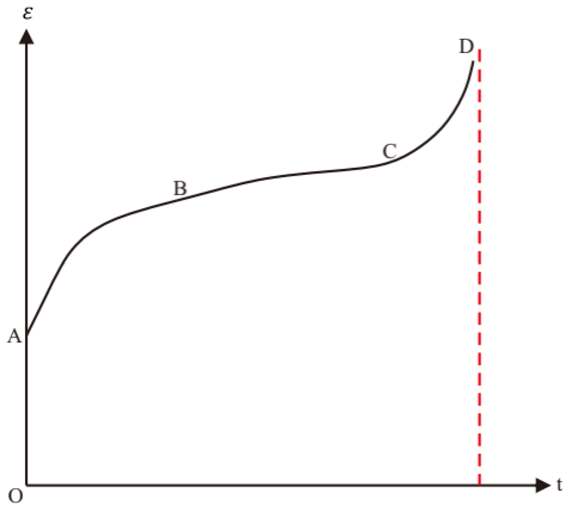
Schematic diagram of creep process.

The creep process could be divided into the three stages based on the creep rate: attenuation stage (AB section), steady stage (BC section), and acceleration ones (CD section) (Fig. 7). When it enters the accelerated creep stage, the deformation rate and creep rate increase obviously (Fig. 7). Nishihara model could describe the initial and steady creep stage, but not the accelerated ones. An elastic body is generally applied to characterize the instantaneous elastic behaviour under loading. During creep load, the deformation property complies with Hooker’s law, considering that the deformation is only related to stress but not to time, and the corresponding constitutive equation is $\sigma =E\dot \varepsilon$. The viscoelastic behaviour of rocks is characterised by a viscous body, whose mechanical properties conform to the definition of a Newtonian fluid, namely, stress proportional to the strain rate, with the constitutive $\sigma =\eta \times \frac{d\varepsilon }{dt}=\eta \dot{\varepsilon }$.

**Figure 8:**
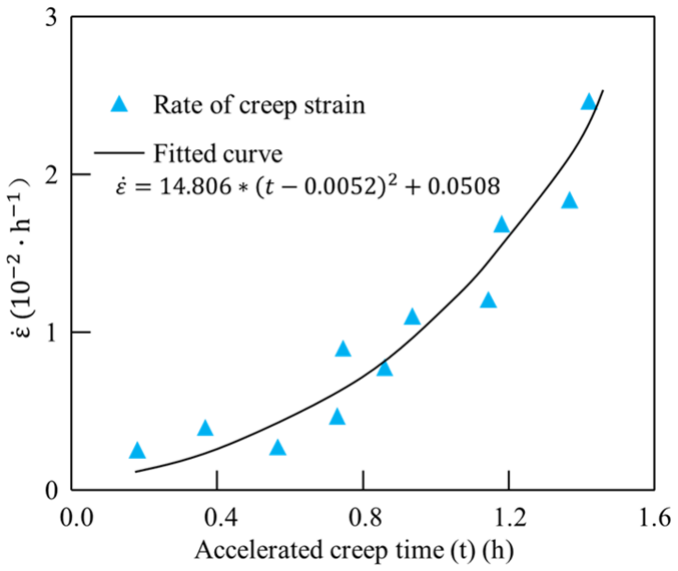
Variation trend of creep rate in accelerated creep stage.

**Figure 9:**
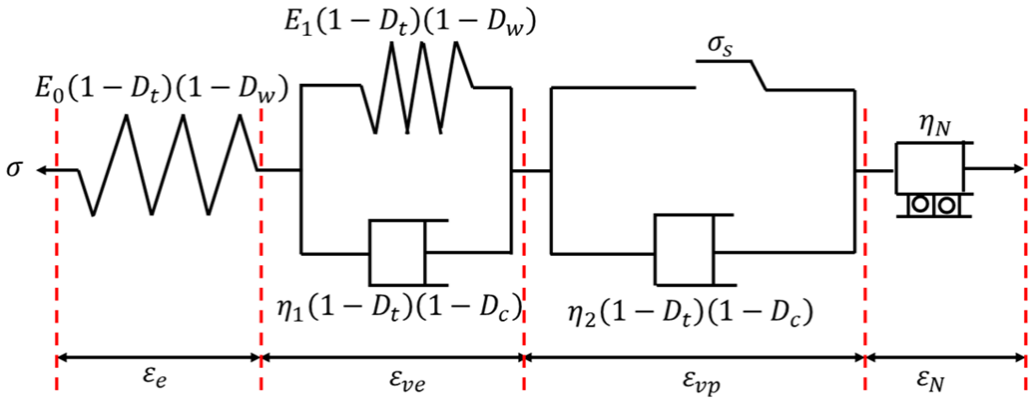
Improved Nishihara model.

Thus, based on the internal relationship between stress and strain or strain rate, a new element was established to describe the accelerated creep behaviour. By analysing the experimental data of the accelerated creep failure stage, the strain rate of the mud shale in the accelerated creep stage was fitted (Fig. 8). It could be seen that, the strain rate $d\varepsilon /dt$ increased approximately exponentially with time, which can be fitted with a quadratic function, continually deriving $d\dot{\varepsilon }/dt$, and finally, finding that $\ddot{\varepsilon }$ increases linearly with time. Under this condition, a new nonlinear viscous pot model with strain triggering is established to describe the accelerated creep process. It only functions only when reaching the accelerated creep stage, which is characterised by a strain greater than ${\varepsilon }_{N}$. When the whole strain of the model is less than ${\varepsilon }_{N}$, the viscous pot could not not work, as shown in Fig. 9. Based on the ideal viscosity theory, a viscous pot is defined as one with stress proportional to $d\ddot{\varepsilon }/dt$, independent of time.

9 $$ \sigma = \eta_{N} \frac{{d\ddot{\varepsilon }}}{dt} = \eta_{N} \dddot \varepsilon $$

In this study, a new creep model is built in series with the viscous pot model and Nishihara model, the improved Nishihara model (Fig. 9). Thus, the total strain of the improved Nishihara model could be expressed as following:

10 $$ \varepsilon = \varepsilon_{e} + \varepsilon_{ve} + \varepsilon_{vp} + \varepsilon_{N} $$

where ${\varepsilon }_{e}$, ${\varepsilon }_{ve}$, ${\varepsilon }_{vp}$, ${\varepsilon }_{N}$ are the elastic, viscoelastic, viscoplastic, and acceleration strains, respectively.

The whole creep equation is expressed:

11 $$  \left\{ \begin{aligned}    & \sigma  = \sigma _{1}  = \sigma _{2}  = \sigma _{3}  = \sigma _{4}  \\     & \sigma _{e}  = E_{0} \left( {1 - D_{t} } \right)\left( {1 - D_{w} } \right)\varepsilon _{1}  \\     & \sigma _{{ve}}  = E_{1} \left( {1 - D_{t} } \right)\left( {1 - D_{c} } \right)\varepsilon _{2}  + \eta _{1} \left( {1 - D_{t} } \right)\left( {1 - D_{c} } \right)\dot{\varepsilon }_{2}  \\     & \sigma _{{vp}}  = \eta _{2} \left( {1 - D_{t} } \right)\left( {1 - D_{c} } \right)\dot{\varepsilon }_{2}  \\     & \sigma _{N}  = \eta _{N} \dddot \varepsilon _{4}  \\  \end{aligned}  \right. $$

where ${\sigma }_{1}$, ${\sigma }_{2}$, ${\sigma }_{3}$, ${\sigma }_{4}$ are the stresses, when each element in the model is connected in series, $\sigma$ is the total stress, and $\varepsilon$ is the total strain. Here, *E*_0_ and *E*_1_ are the elastic coefficients of the spring. ${\eta }_{1}$ and ${\eta }_{2}$ are the viscosity coefficients of the viscoplastic body. ${\sigma }_{s}$ is the mud shale yield stress of the creep. *D*_*w*_ is the hydration damage variable. *D*_*c*_ is the damage variable of the creep viscosity coefficient. *D*_*t*_ is the aging damage variable.

The aforementioned study found that the elastic damage coefficient only acted on the elastic components in the model, whereas the viscosity coefficient acted on the viscous components in the model. By Laplace transform:

12 $$ \varepsilon \left( s \right) = \varepsilon_{e} \left( s \right) + \varepsilon_{ve} \left( s \right) + \varepsilon_{vp} \left( s \right) + \varepsilon_{N} \left( s \right) = \frac{\sigma }{{E_{0} \left( {1 - D_{t} } \right)\left( {1 - D_{w} } \right)s}} + \frac{\sigma }{{\left[ {E_{1} \left( {1 - D_{t} } \right)\left( {1 - D_{c} } \right) + \eta_{1} \left( {1 - D_{t} } \right)\left( {1 - D_{c} } \right)s} \right]s}} + \frac{{\sigma - \sigma_{s} }}{{\eta_{2} \left( {1 - D_{t} } \right)\left( {1 - D_{c} } \right)s^{2} }} + \frac{\sigma }{{\eta_{N} s^{4} }} $$

The creep constitutive equations for different creep stages were achieved with Laplace inverse transformation, which was expressed as follows:

13 $$ \sigma + \frac{{\eta_{1}^{*} }}{{E_{0}^{*} + E_{1}^{*} }}\dot{\sigma } = \frac{{E_{0}^{*} E_{1}^{*} }}{{E_{0}^{*} + E_{1}^{*} }}\varepsilon + \frac{{E_{0}^{*} \eta_{1}^{*} }}{{E_{0}^{*} + E_{1}^{*} }}\dot{\varepsilon },\;\;\sigma < \sigma_{s} $$

14 $$ \ddot{\sigma } + \frac{{\left( {E_{0}^{*} \eta_{2}^{*} + E_{1}^{*} \eta_{2}^{*} + E_{1}^{*} \eta_{1}^{*} } \right)}}{{\eta_{1}^{*} \eta_{2}^{*} }}\dot{\sigma } + \frac{{E_{0}^{*} E_{1}^{*} }}{{\eta_{1}^{*} \eta_{2}^{*} }}\sigma = E_{1}^{*} \ddot{\varepsilon } + \frac{{E_{0}^{*} E_{1}^{*} }}{{\eta_{1}^{*} }}\dot{\varepsilon },\;\;\sigma \ge \sigma_{s} \;{\text{and}}\;\varepsilon < \varepsilon_{c} $$

15 $$ \varepsilon = \frac{\sigma }{{E_{0} \left( {1 - D_{t} } \right)\left( {1 - D_{c} } \right)}} + \frac{\sigma }{{E_{1} \left( {1 - D_{t} } \right)\left( {1 - D_{c} } \right)}}\left\{ {1 - {\text{exp}}\left[ { - \frac{{E_{1} \left( {1 - D_{w} } \right)}}{{\eta_{1} \left( {1 - D_{c} } \right)}}t} \right]} \right\} + \frac{{\sigma - \sigma_{s} }}{{\eta_{2} \left( {1 - D_{t} } \right)\left( {1 - D_{c} } \right)}}t + \frac{\sigma }{{6\eta_{N} }}\tau^{3} ,\;\sigma \ge \sigma_{s} \;{\text{and}}\;\varepsilon > \varepsilon_{N} $$

where $E_{0}^{*} = E_{0} \left( {1 - D_{t} } \right)\left( {1 - D_{w} } \right),E_{1}^{*} = E_{1} \left( {1 - D_{t} } \right)\left( {1 - D_{c} } \right),\eta_{1}^{*} = \eta_{1} \left( {1 - D_{t} } \right)\left( {1 - D_{c} } \right),\eta_{2}^{*} = \eta_{2} \left( {1 - D_{t} } \right)\left( {1 - D_{c} } \right)$; $\tau$ is accelerated creep time; $\tau = t - t_{{\varepsilon = \varepsilon_{N} }}$.

### Determination of parameters and verification of model

**Figure 10:**
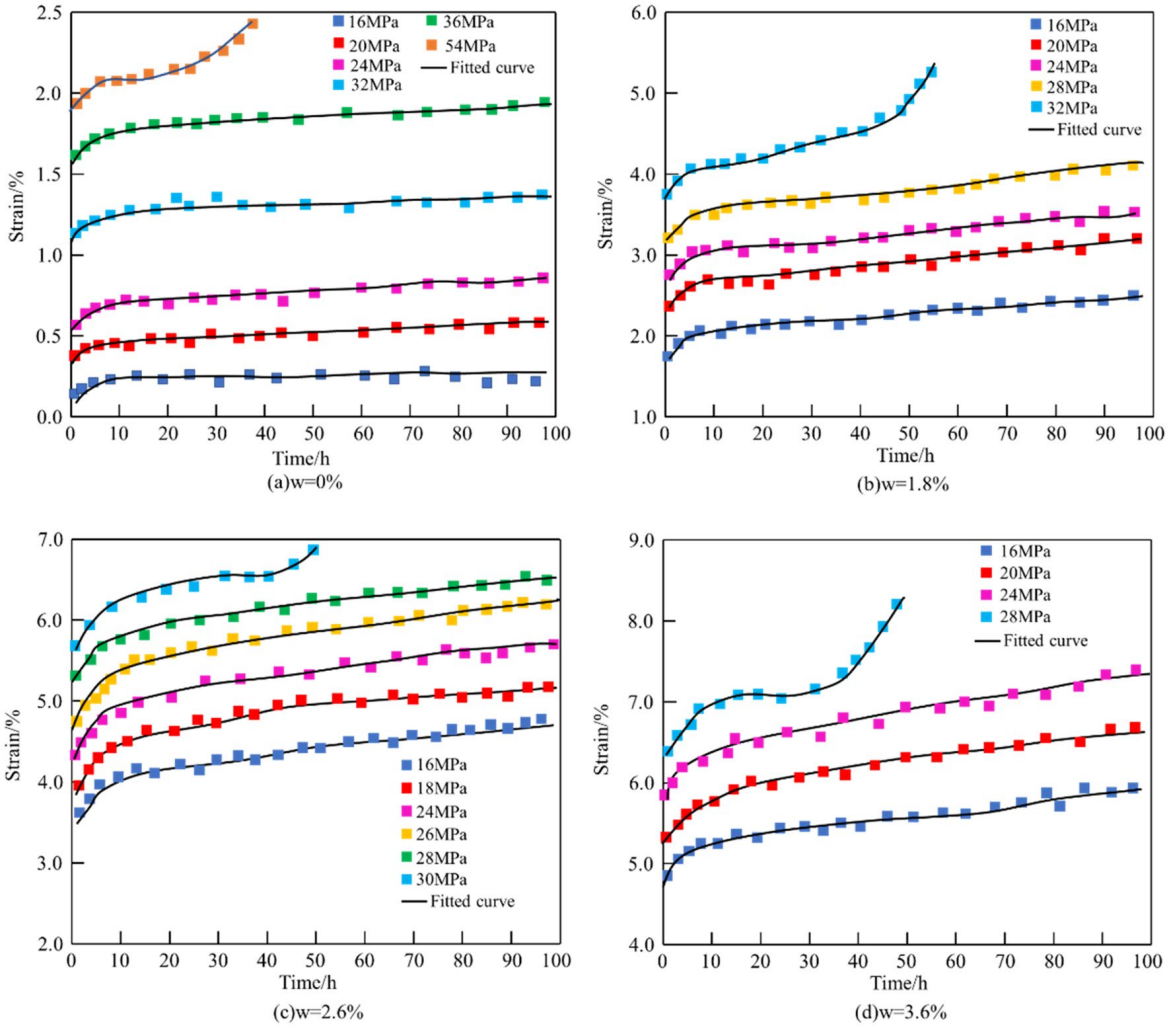
Comparison between creep test curve and simulated curve with an established model.

By analysing the experimental creep data of mud shale with different moisture contents in the western China, the time at which creep reached the accelerated creep stage was determined. The determining process of parameters was as follows: First, obtaining the damaged viscoelastic element parameters ${E}_{0}^{*}$, ${E}_{1}^{*}$, ${\eta }_{1}^{*}$, ${\eta }_{2}^{*}$ by fitting the curves of the first two stages; subsequently, determing the accelerated creep parameter ${\eta }_{N}$ by fitting the curves of the accelerated creep stage after substituting the parameters obtained earlier into the equation; after these two steps, the Levenberg–Marquardt nonlinear least squares method was adopted in this study to obtain the damage creep model parameters under different moisture contents, as shown in Table 3. Figure 10 shows the comparison results of the uniaxial creep experimental curve by step loading and the damaged creep model curve of mud shale with moisture contents of 0%, 1.8%, 2.6%, and 3.6%. The experimental curve is in line with the theoretical curves, which shows that this model can not only accurately describe the entire creep process, including the initial, steady state, and accelerated creep, but can also describe the creep characteristics under different moisture contents. Therefore, the new model could characterise the creep law of mud shale under different moisture contents.

**Table 3:** Fitted parameters of nonlinear damage creep model.

Moisture content (%)	Creep stress (MPa)	Model parameter				
		$${E}_{0}^{*} $$(GPa)	$${E}_{1}^{*}$$ (GPa)	$${\eta }_{1}^{*} $$ (GPa$$ h$$)	$${\eta }_{2}^{*} $$ (GPa$$ h$$)	$${\eta }_{N} $$ (GPa$$ h$$)
0.0	16	5.62	170.27	751.83		
	20	6.53	126.38	569.92		
	24	7.16	89.09	393.58		
	28	8.02	54.53	301.49		
	32	8.79	49.31	229.86		
	36	13.41	47.59	196.71	1460.52	
	54	12.59	43.11	158.82	1268.51	6.96
0.6	16	5.22	211.01	850.22		
	20	5.91	119.02	400.12		
	24	6.49	74.86	326.87		
	28	7.12	53.02	273.59		
	32	7.53	48.75	216.06	85.92	0.06
1.8	16	6.31	187.92	879.90		
	20	7.43	141.76	565.79		
	24	8.11	119.71	379.41	2869.08	
	28	8.92	107.86	319.66	2245.88	
	32	9.53	96.43	199.06	1250.73	15.36
2.6	16	6.11	209.27	855.09		
	18	6.32	185.56	795.28		
	20	6.83	162.08	569.33	1830.55	
	22	7.19	143.10	376.06	1450.39	
	24	7.62	119.37	329.21	1172.43	
	26	7.83	93.11	290.54	976.38	
	28	8.53	79.82	269.67	893.59	
	30	8.63	69.01	258.22	490.31	0.06
3.6	16	5.11	173.81	839.26		
	20	5.68	123.09	450.31	2565.70	
	24	6.20	106.62	322.49	1450.33	
	28	6.68	51.13	147.61	163.83	0.09

#### Application

Based on the established creep model, the equation of drilling fluid density can be deduced. The relationship between drilling fluid density and wellbore shrinkage ratio can be obtained (Eq. 16), which provides a reference for determining a reasonable mud density in drilling engineering.

16 $$ \rho = \frac{100}{H}\left[ {P_{0} - \frac{\sqrt 3 }{3}nR^{2} E_{1}^{*} {\text{exp}}\left( {\frac{{E_{1} }}{{\eta_{1} }}t} \right) - \frac{1}{{2R^{2} }}{ }} \right] $$

where ${\mathrm{P}}_{0}=\left({\upsigma }_{\mathrm{H}}+{\upsigma }_{\mathrm{h}}\right)/2$;R is the wellbore radius; H is the formation depth.

**Figure 11:**
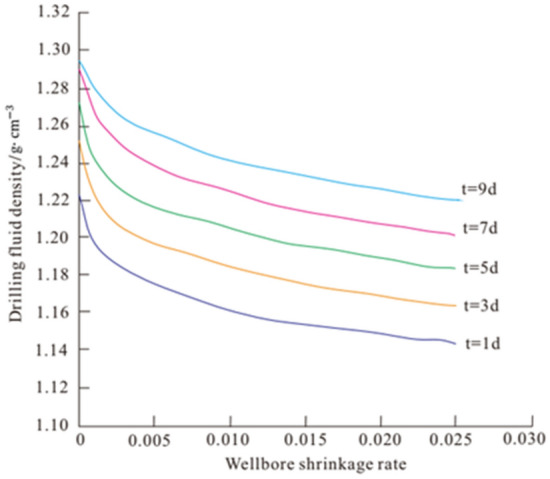
Relation between drilling fluid density and wellbore shrinkage ratio.

This drilling fluid density formula was applied to Well X211 in western China, and the required parameters are listed in Table 4. The density map required for drilling engineering under different well shrinkage rates in this formation is shown in Fig. 11. As can be seen in Fig. 11, the lower the shrinkage rate of the wellbore, the higher should be the density of the drilling fluid; moreover, the longer the drilling time, the higher should be the density of the drilling fluid. The drilling fluid density must be greater than 1.16 g/cm^3^ to keep the wellbore shrinkage rate below 3%. Therefore, the mud density can be reasonably configured according to the change in shrinkage rate caused by creep.

**Table 4:** Formation parameters.

Well depth (H/m)	Wellbore radius (r/mm)	P_0_/MPa	Cohesion (C/MPa)	Poisson’s ratio ($\mu$)	Elastic modulus (E/GPa)
2525	311	53.7	28.9	0.23	33.67

## Conclusions

1. The rock strength of the mud shale was significantly damaged by the increase in moisture content. Moreover, the elastic modulus decreased with increasing water content.
2. Mud shale exhibits prominent creep characteristics. The creep process can be divided into attenuation creep, steady creep, and accelerated creep stages.
3. A new, improved creep model based on the Nishihara model was established to describe the accelerated creep characteristics of mud shale under different moisture contents. The ageing degradation and water-bearing weakening effects were introduced. The creep curve simulated by the model is in line with the experimental results, which indicates that the model is correct and can reliably determine the mud density.

## Data Availability

The datasets generated and/or analysed during the current study are not publicly available because of the requirements of the laboratory but are available from the corresponding author upon reasonable request.
